# Advantage of a Narrow Spectrum Host Defense (Antimicrobial) Peptide Over a Broad Spectrum Analog in Preclinical Drug Development

**DOI:** 10.3389/fchem.2018.00359

**Published:** 2018-08-21

**Authors:** Eszter Ostorhazi, Ralf Hoffmann, Nicole Herth, John D. Wade, Carl N. Kraus, Laszlo Otvos Jr.

**Affiliations:** ^1^Institute of Medical Microbiology, Semmelweis University, Budapest, Hungary; ^2^Institute of Bioanalytical Chemistry, Leipzig University, Leipzig, Germany; ^3^Florey Institute of Neuroscience and Mental Health, University of Melbourne, Melbourne, VIC, Australia; ^4^School of Chemistry, University of Melbourne, Melbourne, VIC, Australia; ^5^Arrevus, Inc, Raleigh, NC, United States; ^6^OLPE, LLC, Audubon, PA, United States

**Keywords:** *Acinetobacter baumannii*, anti-inflammatory cytokines, ARV-1502, bacteremia model, minimal inhibitory concentration, toxicity

## Abstract

The APO-type proline-arginine-rich host defense peptides exhibit potent *in vitro* killing parameters against *Enterobacteriaceae* but not to other bacteria. Because of the excellent *in vivo* properties against systemic and local infections, attempts are regularly made to further improve the activity spectrum. A C-terminal hydrazide analog of the Chex1-Arg20 amide (ARV-1502) shows somewhat improved minimal inhibitory concentration against *Moraxellaceae*. Here we compared the activity of the two peptides as well as an inactive dimeric reverse amide analog in a systemic *Acinetobacter baumannii* infection. Only the narrow spectrum amide derivative reduced the 6-h blood bacterial burden by >2 log_10_ units reaching statistical significance (*p* = 0.03 at 5 mg/kg and 0.031 at 2 mg/kg administered intramuscularly). The hydrazide derivative, probably due to stronger activity on cell membranes, lysed erythrocytes at lower concentrations, and caused toxic effects at lower doses (10 mg/kg vs. 25 mg/kg). In a limited study, the amide induced a >5-fold production of the anti-inflammatory cytokine IL-10 over untreated naïve mice and minor increases in the anti-inflammatory IL-4 and pro-inflammatory cytokines TNF-α and IL-6, in blood. The blood of hydrazide-treated mice exhibited significantly lowered levels of IL-10 and slightly decreased IL-4 and TNF-α. These results suggest that the improved efficacy of the narrow-spectrum amide analog is likely associated with increased anti-inflammatory cytokine production and better stimulation of the immune system. Although blood IL-6 and TNF-α levels are frequently used as markers of potential toxicity in drug development, we did not observe any notable increase in mice receiving the toxic polyamide antibiotic colistin.

## Introduction

Current antimicrobial research aims to develop broad spectrum agents to help clinicians who must initiate empirical treatment regimen in systemic infections before the pathogens are even identified (Kollef, 2008). This notion comes from retrospective analysis of treatment success rates when small molecule antibiotic therapy is applied in hospitals (Tumbarello et al., 2007). To satisfy USA and European regulatory agencies, the *in vitro* minimal inhibitory concentration (MIC) clinical breakpoints should be below 16 mg/L against almost all pathogens, more preferably below 2–4 mg/L (EUCAST, 2018; US Department of Health Human Services Food Drug Administration, 2018). Antimicrobial peptides (AMPs) can seldom satisfy these recommendations, even if the osmolarity of test media is reduced for improved MIC readouts (Cudic et al., 2003).

Nevertheless, AMPs have been considered powerful drugs against many systemic infections in animal models since the 1990s (Hancock and Lehrer, 1998). A growing body of literature has demonstrated that AMPs have a plethora of activities on both bacteria and the hosts, immunostimulatory effects being equally, or more important than antimicrobial activity (Brandenburg et al., 2012; Otvos, 2016). Likewise, the innate immune system of the host can be upregulated by external AMP introduction (Knappe et al., 2016). The subsequently coined term, host defense peptide (HDP) (Nijnik and Hancock, 2009) is especially true for the proline-arginine-rich family of AMP, whose *in vivo* efficacy simply cannot be correlated with *in vitro* bacterial killing in topical infections (Ostorhazi et al., 2011a, 2013). The proline-arginine-rich HDP dimer A3-APO sterilizes wounds and intradermal tissues infected with *Staphylococcus aureus* or *Propionibacterium acnes* despite having no measurable *in vitro* MIC against these pathogens. The monomeric version of A3-APO, referred to as Chex1-Arg20 or ARV-1502, has even greater efficacy in *P. acnes* (administered topically) or *Acinetobacter baumannii* systemic infection models (Ostorhazi et al., 2011b, 2013), in spite of high MIC values and having limited effects on bacterial membranes, both of which are drivers of broad spectrum *in vitro* functions (Cassone et al., 2008; Li et al., 2016).

The Chex1-Arg20 amide (ARV-1502) peptide is currently in development as a therapeutic measure against bacterial peritonitis. During our search for the optimal analog to enter pharmaceutical development, we compared the *in vivo* efficacy and toxicity parameters of a hydrazide C-terminal derivative of the same sequence. The hydrazide version was shown to have slightly reduced activity against sensitive *Enterobacteriaceae* including *Escherichia coli* and *Klebsiella pneumoniae* but improved MIC values against two largely resistant Gram-negative species, *Acinetobacter baumannii* (*Moraxellaceae*) and *Pseudomonas aeruginosa* (*Pseudomonadeceae*). Although the infectious agent in the current report was *A. baumannii*, the narrow spectrum Chex1-Arg20 amide outperformed the hydrazide in reducing the blood bacterial burden probably because of increased immunostimulatory activities (as measured by anti-inflammatory IL-10 production) compared to the broad spectrum analog.

## Materials and methods

### peptide synthesis

The peptides used in the current report were synthesized previously for other studies. Table 1 lists the peptides and their first publication references. All peptides were purified by reversed-phase high-performance liquid chromatography (RP-HPLC), characterized by matrix-assisted laser desorption/ionization mass spectroscopy, dialyzed twice from 1% acetic acid (to exchange trifluoroacetate counterions to acetate) and the actual peptide content was quantified by amino acid analysis or RP-HPLC.

**Table 1:** Peptides studied in the current study.

	**Sequence**	**Original description**
Chex1-Arg20 amide	H-Chex-Arg-Pro-Asp-Lys-Pro-Arg-Pro-Tyr-Leu-Pro-Arg-Pro-Arg-Pro-Pro-Arg-Pro-Val-Arg-NH_2_	Noto et al., 2008
Hydrazide analog	H-Chex-Arg-Pro-Asp-Lys-Pro-Arg-Pro-Tyr-Leu-Pro-Arg-Pro-Arg-Pro-Pro-Arg-Pro-Val-Arg-NH-NH_2_	Li et al., 2015
Reverse	H-Chex-Arg-Val-Pro-Arg-Pro-Pro-Arg-Pro-Arg-Pro-Leu-Tyr-Pro-Arg-Pro-Lys-Asp-Pro-Arg-NH_2_	Li et al., 2017a
A3-APO dimer	(H-Chex-Arg-Pro-Asp-Lys-Pro-Arg-Pro-Tyr-Leu-Pro-Arg-Pro-Arg-Pro-Pro-Arg-Pro-Val-Arg)_2_-Dab	Otvos et al., 2005

*The monomeric APO (All Peptides Optimized, Otvos et al., 2005) variants are referred to in the literature as monomer or ARV-1502 (Chex1-Arg20 amide), hydrazide monomer and reverse. The peptides were not separately synthesized for this study; rather the preparations for the indicated publications were used*.

### Measurement of minimal inhibitory concentration

MIC were measured in a liquid broth microdilution assay using sterile 96-well plates (Krizsan et al., 2015). Aqueous peptide solutions were serially twofold diluted (512 to 0.06 mg/L) in 25% or undiluted Muller-Hinton broth (MHB) medium and mixed with overnight cultures diluted in the same media to 1.5 × 10^7^ cells/mL. The total volume was 100 μL per well. The plates were incubated for 20 ± 2 h at 37°C. Optical density was measured at 595 nm. The MIC was defined as the lowest peptide concentration at which the turbidity reading did not exceed that of medium only.

#### Lysis of red blood cells

Blood was collected from a 47-year old healthy donor (EO). The plasma was discarded and the remaining cells were diluted in physiological salt solution 100-fold. To 500 μL of this suspension were added 50 μL of test peptides or 1% (w/v) Triton X-100 dissolved in distilled water. The peptide concentrations were: 100, 200, and 400 mg/L. The vials were incubated at 37°C for 2 h, the cells were centrifuged at 6,000 rpm for 1 min, and the vials were photographed.

### Animals

Three and a half-to-four and a half-week old female NMRI (Naval Medical Research Institute) BR mice (Toxi-Coop Zrt, Budapest, Hungary) were used throughout these studies. The mice were housed in plastic type 2 individually ventilated cages, 3 mice per cage, on softwood granules as bedding. The room was kept between 21 and 25°C with 12 h light: 12 h dark cycles. The animals had free access to tap water and pelleted rodent food. Upon completion of the experiments, surviving mice were euthanized by diethyl ether inhalation. Animals were maintained and handled in accordance with the recommendations of the Guidelines for the Care and Use of Laboratory Animals, and the protocols were approved by the Animal Care Committee of Semmelweis University (permission No.: PE/EA/60-8/2018).

#### Infection model

Mice weighing ~17–20 g (4 weeks old) were randomly divided into 6 groups, 5 mice in each group, and infected by intraperitoneal (ip) injection of 4.8 McFarland (2 × 10^7^ CFU/g) *A. baumannii* BAA-1605 (Ostorhazi et al., 2017). Peptides were administered intramuscularly (im) at 5 or 2 mg/kg doses in phosphate buffered saline (PBS) 1 h after infection. Blood (10 μL) was taken 6 h after infection for blood bacterial count determination. The blood was prevented from coagulation with ethylene diamine tetraacetic acid and the samples were serially diluted in 0.9% saline. Each dilution was cultured providing a detectable threshold of 10^3^ CFU/mL. Survival was monitored at 24 h after infection.

Blood bacterial load reduction in the various groups was compared with unpaired Student's t-testing (SlideWrite, Encinitas, California, USA).

#### *In vivo* toxicity

Mice (5 per group) were weighed before peptide administration. Peptides were administered im at 10, 25, 50, 75, and 100 mg/kg doses. Mice were observed for behavioral effects for 24 h (Ostorhazi et al., 2010). Toxicity levels were identified as 1, transient low-medium effects (reduced activity, cuddling, shivering) and 2, transient serious effects (complete lack of movements). After 24 h, the mice were weighed again, ethically euthanized and gross necropsied for signs of obvious organ damage changes.

### Cytokine quantitation

NMRI mice were inoculated with the Chex1-Arg20 amide and hydrazide peptides in doses 2, 5 or 10 mg/kg (one mouse for each dose and antibiotic for pro- and one mouse per dose and antibiotic for anti-inflammatory cytokines), the serum was collected after 24 h and was analyzed in duplicates for IL-4, IL-10, IL-6, and TNF-α content by using ELISA kits available for this purpose (Cedarlane Labs, Burlington, Ontario, Canada). Briefly, the sera and cytokine standards were loaded onto pre-coated 24 well (IL-4) or 96-well (IL-10, IL-4, and TNF-α) plates, and the plates were reacted with biotinylated antibodies specific for the given cytokines. The cytokine concentration was quantified by using streptavidin-horseradish peroxidase and tetramethyl benzidine substrate solutions. Absorbance was read at 450 nm. For IL-6 and TNF-α determination, the test samples included blood of mice receiving 30 mg/kg imipenem or 10 mg/kg colistin subcutaneously (sc) both doses calculated as the allometrically scaled human clinical doses. The breadth of the assay was designed to fit all calibrating and test samples to plates pre-coated for each cytokine.

## Results

### MIC measurements

In previous studies, the Chex1-Arg20 amide (ARV-1502) was shown to inhibit the growth of *E. coli* and *K. pneumoniae* strains with ~2-fold reduction in MIC values (6.3 vs. 10 mg/L and 2 vs. 4.2 mg/L respectively) compared to the hydrazide analog (Li et al., 2015, 2017a). At the same time, the hydrazide was twofold more active than the amide against *A. baumannii* and *P. aeruginosa* (130 vs. >250 mg/L and 72 vs. 140 mg/L, respectively). The reverse peptide (Table 1) was completely inactive against even the otherwise sensitive strain *K. pneumoniae* (Li et al., 2017b). These results were observed by using the Lambert and Pearson growth curve analysis method (Lambert and Pearson, 2000), in which the MIC is provided by extrapolation of maximal growth against the antibiotic concentration. Regulatory agencies prefer no bacterial growth as MIC readout and thus we repeated the assays with a traditional broth microdilution assay in full and 25% MHB media. In the current study, the amide retained the ~2-fold improvement in MIC values compared to the hydrazide against *K. pneumoniae* but was no less active than the hydrazide against *A. baumannii* (Table 2). Of note, the MIC remained above the reported MIC values even in quarter-strength MHB. The studied *A. baumannii* strain with 64 mg/L MIC value can be considered resistant to both peptides. Our current studies verified the very weak or almost no *in vitro* activity of the reverse derivative (Table 2).

**Table 2:** Minimal inhibitory concentrations (MIC) of the APO peptide analogs against Gram-negative bacteria.

**Antibiotic**	**MIC (mg/L) in 25% MHB**	**MIC (mg/L) in full MHB**	**MIC (mg/L) in 25% MHB**	**MIC (mg/L) in full MHB**
	***K. pneumoniae***	***K. pneumoniae***	***A. baumannii***	***A. baumannii***
Chex1-Arg20 amide	4	128 (2)	64	>512 (>250)
Chex1-Arg20 hydrazide	8-16	256 (4.2)	64	>512 (130)
reverse amide	32-64	>512 (n.t.)	64	>512 (n.t.)
Meropenem	0.06	0.1	0.5	4

*The test Klebsiella pneumoniae strain is designated as K. pneumoniae 1102, and the Acinetobacter baumannii strain is designated as A. baumannii 30008. The values in parentheses show the reported activity of the same peptides against K. pneumoniae ATCC 13883 and A. baumannii ATCC 19606 (Li et al., 2015; n.t = not tested). Please note the differences in the MIC readout methodology of the two different assay protocols. In the current study K. pneumoniae represented sensitive and A. baumannii represented resistant bacteria for the Chex1-Arg20 amide (ARV-1502). In the Li paper the hydrazide derivative of Chex1-Arg20 was twice as active as the parent amide analog against P. aeruginosa ATCC 4705 (MIC = 72 vs. 140 mg/L). In the current test assay meropenem was used as a positive control*.

### Reduction of bacterial load in a systemic mouse *A. baumannii* infection model

**Figure 1:**
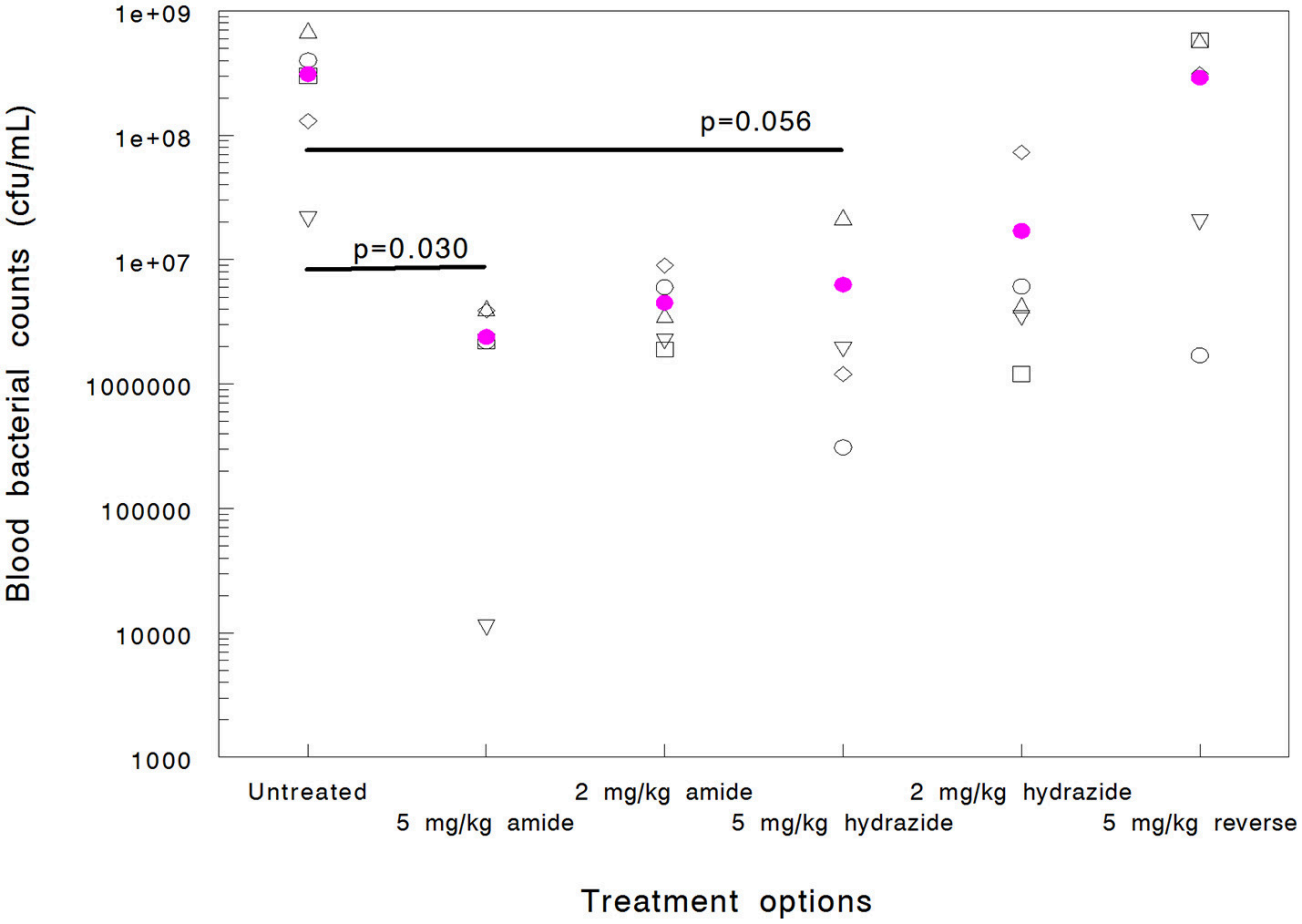
Efficacy of APO peptide analogs in a mouse systemic *Acinetobacter baumannii* infection model as documented by blood bacterial counts (cfu/mL) measured 6 h after infection (5 h after treatment). Each group contained 5 randomly selected animals. Statistically significant reductions were observed for the Chex1-Arg20 amide (ARV-1502) at 5 mg/kg (*p* = 0.030) and 2 mg/kg (*p* = 0.031) doses. Hydrazide analog treatment did not yield statistically significant improvement (*p* = 0.056 at 5 mg/kg and 0.068 at 2 mg/kg). In addition, one mouse in the 5 mg/kg hydrazide analog-treated group died before the 6-h blood sampling (thus blood bacteria could not be enumerated). The reverse peptide was inactive in this measure.

When mice are infected with *A. baumannii* 1605 and dosed with Chex1-Arg20 amide (ARV-1602) three times intramuscularly, the peptide prevents mortality in >50% of the animals in spite of having no or minimal killing ability to this strain *in vitro* (Ostorhazi et al., 2011b). Here we used the same model to compare the *in vivo* protective effects of the Chex1-Arg20 analogs, except that the mice received only 1 dose of peptide to better compare the blood bacterial counts early (6 h) in the disease progression. If directly killing bacteria, AMPs are reported to act rapidly both *in vitro* and *in vivo* (Zasloff, 2002; Mohamed et al., 2016) and this is especially true for proline-arginine-rich peptides (Holfeld et al., 2017, 2018). Six hours after infection (5 h after peptide administration) the blood bacterial counts of untreated mice elevated to 3.1 × 10^8^ CFU/mL (Figure 1). Mice treated with the reverse peptide exhibited no decrease in blood CFU levels (2.9 × 10^8^ CFU/mL). The Chex1-Arg20 amide and hydrazide peptides reduced the blood bacterial load in a dose-dependent manner. At 2 mg/kg hydrazide treatment the blood bacterial load was reduced to 1.7 × 10^7^ CFU/mL and at 5 mg/kg to 6.3 × 10^6^ CFU/mL (Figure 1). However, neither of these values reached statistical significance. Statistically significant reduction in blood bacterial load was reached by using the amide version at both doses; 2 mg/kg resulting in 4.5 × 10^6^ CFU/mL (*p* = 0.031) and 5 mg/kg resulting in 2.4 × 10^6^ CFU/mL (*p* = 0.030).

While this study was not designed to monitor differences in survival (the single low dose therapy measure was not expected to markedly improve survival), we did find that peptide amide treatment resulted in a higher number of surviving animals early in the treatment course when dosed at 5 mg/kg (100% vs. 80% at 6 h), and at the lower dose later in the study (20% vs. 0% at 24 h dosed at 2 mg/kg). Taken together, there was a signal that the Chex1-Arg20 amide version (ARV-1502) was more active in the animal model than the hydrazide analog, in spite of being a narrower spectrum derivative *in vitro* and not killing the test strain in the MIC measure.

### Toxicity

Since the A3-APO dimer's broadened *in vitro* efficacy compared to the monomer amide is due to stronger effects on bacterial membranes (Cassone et al., 2008; Li et al., 2016), we hypothesized that the hydrazide similarly exhibits better MIC values against resistant strains because of increased activity on membranes. As broad spectrum AMPs are usually increasingly toxic (Bush et al., 2004), we wondered whether the hydrazide version is more toxic to mice, a phenomenon that could partially explain the lowered efficacy figures. Mice were inoculated im with 10, 25, 50, 75, and 100 mg/kg doses of peptides once. We monitored the weight gains and behavioral effects for 24 h. Amide-treated peptides gained weight during the assay period, with lower doses resulting in higher weight increases (Figure 3). While the weight increases at 10 and 25 mg/kg were in the range of those of untreated animals, mice treated with higher doses gained less weight than normal female NMRI mice of similar age. The hydrazide analog-treated mice gained less weight when dosed at 10 and 25 mg/kg and lost increasingly more body mass at 50, 75, and 100 mg/kg peptide treatment. Clearly, the hydrazide analog appears to be more toxic than the amide version. In addition, while hydrazide analog-treated mice showed no visible signs of discomfort only at the 10 mg/kg dose, amide-treated mice had no discomfort at 10 and 25 mg/kg doses. All mice quickly recovered from toxicity symptoms and returned to normal activity levels within 2 h.

**Figure 2:**
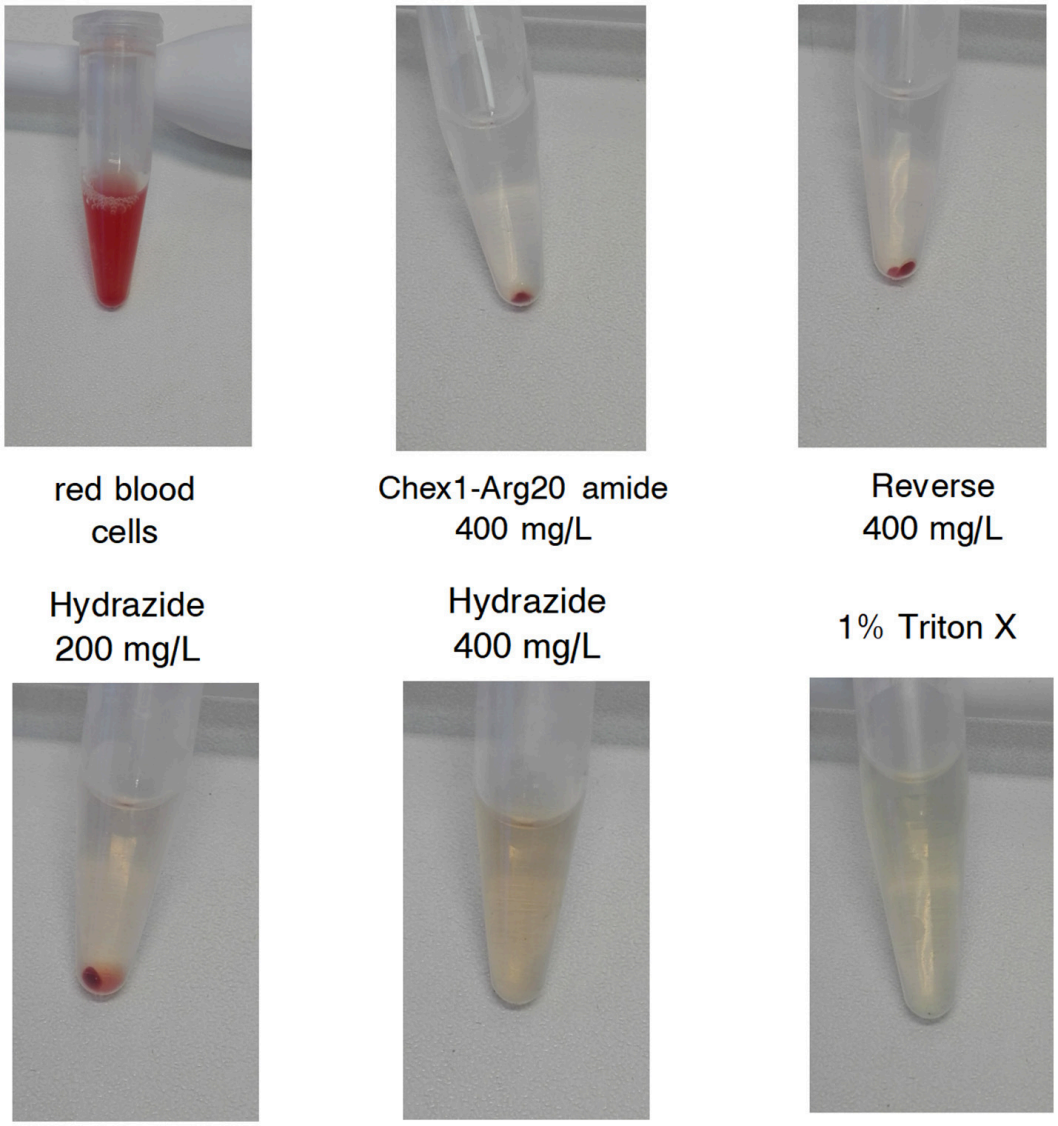
Effect of APO monomeric peptides to a 1% (v/v) human red blood cell (RBC) suspension. Top left: untreated RBC suspension. Top middle, top right, bottom left, and bottom middle: the suspension was treated with the given concentration of the peptides for 2 h and the cells were centrifuged. Bottom right: cells treated with 1% Triton X-100. The highest concentration of the hydrazide analog and the positive control surfactant dissolved the cells; the Chex1-Arg20 amide (ARV-1502) and reverse derivatives had no effect on RBC.

The increased *in vivo* toxicity of the hydrazide analog should manifest in lysis of red blood cells at lower concentrations *in vitro*. Both peptides were reported not to have any effects on rat hepatoma and human embryonic kidney cells at 250 mg/L (Li et al., 2015, 2017b). However, at an MIC of 130 mg/L (converted from μM concentrations in the published reports), the therapeutic index (TI) would only be 2. Here we increased the peptide concentration to 400 mg/L and were looking at effects on red blood cells from a healthy human donor. After incubation with peptides, the 1% red blood cell suspensions were photographed. The reverse and the amide analogs did not lyse the cells from any of the 100–400 mg/L concentration range (Figure 2 shows the vials containing 400 mg/L peptides). In agreement with earlier studies, the hydrazide analog had little effect on the cells at 200 mg/L, but in our assay it completely lysed the cells at 400 mg/L (Figure 2). In Figure 2, red blood cells alone and 1% Triton X-100 treated cells represent the negative and positive controls, respectively. In conclusion, when applied in a therapeutically meaningful concentration to identify the TI, the increased toxicity of the hydrazide version observed *in vivo* was verified by *in vitro* measurements.

**Figure 3:**
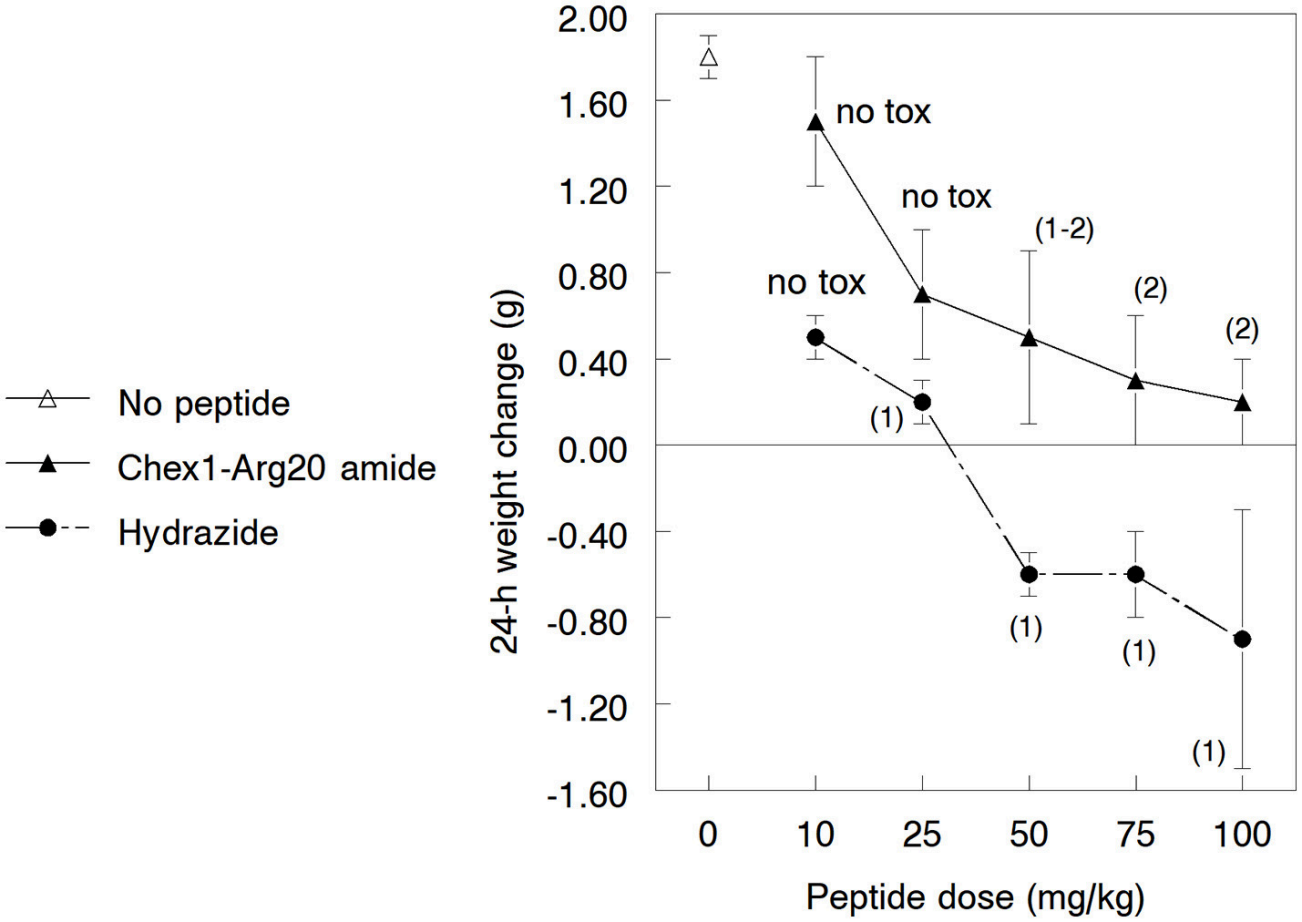
Gross toxicity of the Chex1-Arg20 amide (ARV-1502) and the hydrazide analog to 3.5–4.5 weeks old growing NMRI mice. In every group, 5 mice were treated intramuscularly with the peptides and the weight change was measured after 24 h. During the acclimatization period, the daily weight gain of the mice was 1.6–1.9 g. The usual weight gain of 3.5–4.5 weeks old normal female NMRI mice is 0.9–1.1 g/day. The numbers in parentheses indicate the visual discomfort of the animals: 1, transient low-medium effects—reduced activity, cuddling, shivering; 2, transient serious effects—complete lack of movements. The mice returned to their normal activity patterns 60–90 min after treatment. Mice treated with the amide derivative not only maintained a higher weight than those treated with the hydrazide analog (these actually lost weight at higher doses) but the visual toxic effects started at higher peptide dose regimens.

### Production of anti-inflammatory cytokines

**Figure 4:**
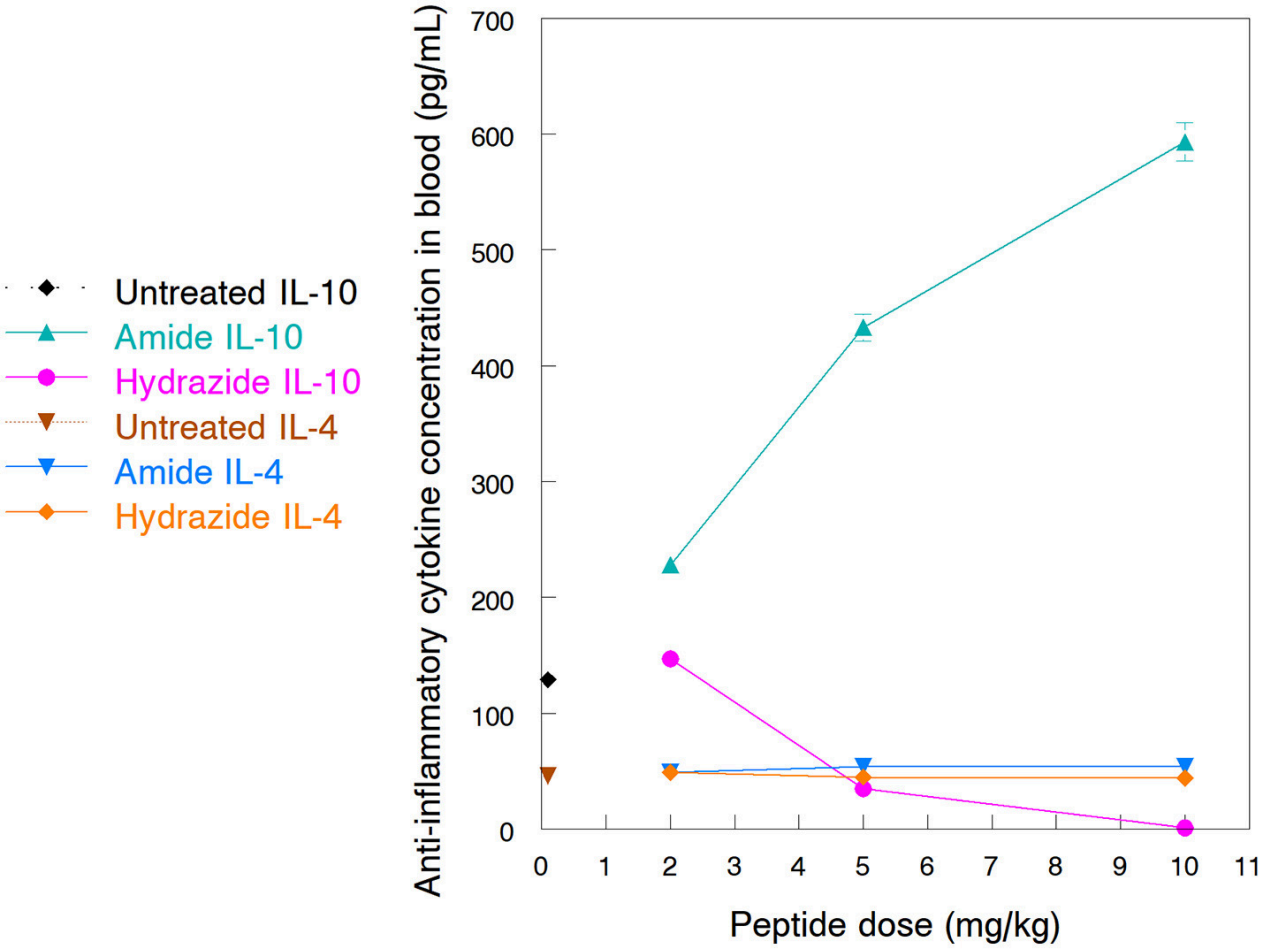
Blood levels of anti-inflammatory cytokines after treatment of NMRI mice with Chex1-Arg20 amide (ARV-1502) and hydrazide peptides. One mouse per antibiotic and dose were intramuscularly (im) injected with 2, 5 and 10 mg/kg peptide doses, and the blood was collected after 24 h. IL-4 and IL-10 concentrations were measured in duplicates with kits available for this purpose. The sample size was designed according to the well limitations of the pre-coated plates.

One of the major mode of actions of HDP is the activation of immune responses, frequently involving anti-inflammatory cytokine production (Brandenburg et al., 2012; Otvos, 2016). Earlier we documented how the A3-APO dimer induces IL-4 and IL-10 production in peripheral blood mononuclear cells (Ostorhazi et al., 2011a). In the current study we compared the increase of IL-4 and IL-10 in mouse blood after treatment with the monomeric amide and hydrazide peptides. The 24-h blood IL-4 content of peptide-treated mice was not significantly different from those receiving physiological salt solution only (Figure 4). If any distinction is to be made, the IL-4 content of mice sera treated with the Chex1-Arg20 amide slightly increased with increasing dose and slightly decreased with increasing dose of the hydrazide. However, even at the highest dose of 10 mg/kg, the IL-4 concentrations differed by only about 10% from those of untreated mice. Significantly higher alterations were observed for the serum IL-10 content. Once again, as the dose of the amide peptide increased, the serum contained increased IL-10 levels, going from 129 pg/mL (untreated) to 228 pg/mL (at 2 mg/kg), 433 pg/mL (5 mg/kg), and 593 pg/mL (at 10 mg/kg). At the 5 mg/kg therapy dose, the blood IL-10 concentration was >3 times of that of untreated mice (Figure 4). Conversely, with increasing the dose of the hydrazide analog to 5 mg/kg and 10 mg/kg, the blood IL-10 decreased to 35 pg/mL, and 1 pg/mL (Figure 4). At the therapy dose in the mouse of 5 mg/kg, the IL-10 content was only about 27% of that of untreated animals.

As some HDP increase pro-inflammatory cytokine levels while others, such as LL-37, can raise both anti- and pro-inflammatory cytokines (Otvos, 2016), we also measured the blood IL-6 and TNF-α content of mice treated with the Chex1-Arg20 amide and hydrazide peptides. The blood TNF-α content of mice receiving no peptide was ~17 pg/mL, the lowest value in the calibrating standard curve. Upon amide-peptide treatment, the TNF-α content was slightly increased (by 2 pg/mL at 2 and 5 mg/kg, and by 6 pg/mL at 10 mg/kg) compared to untreated control animals (Table 3). At the same time points, the hydrazide analog-treated mice had slightly decreased blood TNF-α levels (by 1.5 pg/mL at 2 mg/kg, 2 pg/mL at 5 mg/kg and 5 pg/mL at 10 mg/kg). The blood IL-6 content was less clearly associated with treatment arms. Once again, for Chex1-Arg20 amide-treated mice IL-6 was increased, but increasing doses produced smaller IL-6 elevations (Table 3). Regarding the hydrazide analog-treated mice, the blood IL-6 concentration increased slightly at the lowest dose of 2 mg/kg (3 pg/mL), and decreased at the same level after 5 mg/kg and 10 mg/kg treatments. While no changes were found after 30 mg/kg imipenem treatment, a minor IL-6 increase was observed after exposure to 10 mg/kg colistin (Table 3). While these numbers remain significantly below the IL-10 increases, it was interesting to note that the more active amide analog produced higher levels of all cytokines studied than the less active hydrazide derivative. Allometrically scaled doses of colistin and imipenem induced lower TNF-α and almost unchanged IL-6 levels, comparable to those after Chex1-Arg20 hydrazide treatment (Table 3).

**Table 3:** Changes in blood pro-inflammatory cytokine levels in mice after treatment with antibacterial agents.

**Treatment**	**Concentration of TNF-α (pg/mL) in mouse blood**	**Concentration of IL-6 (pg/mL) in mouse blood**
Untreated control	16.8 ± 0.1	12.4 ± 0
Amide 2 mg/kg im	19.6 ± 0.1	17.1 ± 0.1
Amide 5 mg/kg im	19.9 ± 0.1	16.2 ± 0.1
Amide 10 mg/kg im	23.6 ± 0.1	12.8 ± 0.1
Hydrazide 2 mg/kg im	15.1 ± 0.1	15.1 ± 0.2
Hydrazide 5 mg/kg im	14.7 ± 0	9.5 ± 0
Hydrazide 10 mg/kg im	11.8 ± 0.1	9.2 ± 0.1
Colistin 10 mg/kg sc	13.6 ± 0.1	13.7 ± 0
Imipenem 30 mg/kg sc	12.6 ± 0	11.5 ± 0

*The blood was collected 24 h after and TNF-α and IL-6 content was quantitated in duplicates with kits available for this purpose. One mouse was used for each dose of each antibiotic. Peptides were administered intramuscularly (im) and small molecule antibiotics subcutaneously (sc). The small molecule antibiotic doses correspond to the allometrically scaled human doses in current clinical practice. The allometrically scaled intended therapy dose of the Chex1-Arg20 (ARV-1502) is 5 mg/kg im in mice. The assay size was designed according to the well limitations of the pre-coated plates*.

## Discussion

### *In vitro* activity differences

While the reported activity of the hydrazide analog to *A. baumannii* is considered very weak (MIC = 130 mg/L) (Li et al., 2015, 2017a), even at this low activity level in the current study we could not repeat the superiority of the hydrazide over the amide (64 mg/L and >512 mg/L in 25% and full-strength MHB for both). The reasons for the lack of activity differences might be either alterations in the assay protocol (Lambert and Pearson vs. broth microdilution) or the identity of the actual *A. baumannii* strain. Even by using our standard assay protocol, we regularly observe a 4-fold difference in the MIC values among various *A. baumannii* strains when the assay is run in 25% MHB. Our activity figures against *Moraxellaceae*, with Chex1-Arg20, its dimeric version A3-APO or one of the parent peptide to the APO analogs, pyrrhocoricin, always hover at the limit of detectability (Cudic et al., 2002; Ostorhazi et al., 2011b). Thus, the weak activity of the monomeric amide (this study) or no activity at all (earlier reports) can indeed reflect minor strain or assay protocol differences. In any event, taking all published data into consideration, the amide analog is increasingly active against sensitive strains and the hydrazide analog exhibits a broader (but less potent) activity spectrum.

The reverse peptide was less active than the amide and the hydrazide against the sensitive strain *K. pneumoniae*. One of the major modes of *in vitro* activity of the proline-arginine rich peptides, other than immunostimulation or inhibiting bacterial protein translation (Krizsan et al., 2014), is binding to the 70 kDa heat shock protein DnaK and inhibiting protein folding (Kragol et al., 2001). Measured by fluorescence polarization and dot-blot assays and using synthetic peptide fragments of DnaK, pyrrhocoricin binds both the conventional substrate-bonding pocket and the C-terminal multihelical lid. Binding to the lid appears to be stronger than that to the pocket providing strain and species-selectivity of pyrrhocoricin and its designer daughter variations (Kragol et al., 2001). Based on X-ray diffraction analysis, pyrrhocoricin binds to the substrate-binding pocket in both forward and reverse orientations (Zahn et al., 2013). The lack of any activity of the reverse APO peptide in killing bacteria indicates that binding to the pocket is not responsible for any antimicrobial activity. It is likely that X-ray diffraction captures a snapshot of fast binding under kinetic control whereas, in-solution or mixed solution-membrane techniques reveal longer-term interactions under thermodynamic control, reflective of those processes observable in the presence of whole bacteria.

### *In vitro* and *in vivo* toxicity

Most research reports observe the *in vitro* toxicity of AMPs up to only 100 μM that in our case represents ~250 mg/L. Given MIC values for Chex1-Arg20 against *A. baumannii* of 16–32 mg/L, the range of past *in vitro* toxicity measurements do not even cover a TI of 10. AMPs or HDPs are usually administered in efficacy assays at higher doses than receptor-response modifying peptide drugs. Consequently, the *in vitro* toxicity assays must use higher concentrations than previously to cover reasonable TI ranges and mimic doses injected into the circulation of mice (10 mg/kg, a minimal intravenous (iv) dose of AMP, equals to 200 μg drug injected directly into the 2 mL blood pool of mice or 100 mg/L). Our generally observed iv toxicity level of 10–25 mg/kg (Szabo et al., 2010) is perfectly in line with the calculations presented above. We prefer im dosing to limit high peak concentrations and capitalize on a slow release into the circulation over a period of time (“depot effect,” Ostorhazi et al., 2017), prolonging the exposure of the drug while keeping the maximum blood concentration lower. Indeed, not only proline-arginine-rich peptides such as those presented in the current report have a lower toxicity profile as well as improved activity when administered im, but this observation can be extended to other AMP families as well (Wu et al., 2014).

### Changes in blood cytokine levels

IL-10 is known to downregulate the expression of pro-inflammatory cytokines (Zhang and An, 2007). However, in our hands high levels of IL-10 expression upon peptide treatment did not result in TNF-α or IL-6 expression below of those of untreated animals. In fact, both pro-inflammatory cytokine levels slightly increased after Chex1-Arg20 amide treatment. Potentially, the increased IL-10 levels served for limiting the upregulation of the pro-inflammatory cytokines and thereby suppressing immunopathology (Couper et al., 2008). It is interesting to note that the toxic signs of Chex1-Arg20 we observe in uninfected rats and dogs (Otvos et al., 2018) feature allergic effects and these perhaps signs of efficacy as manifested in IL-10 production rather than tissue toxicity findings.

A number of AMP/HDP families including the defensin and cathelicidin families activate innate immunity *via* pro-inflammatory signaling (Otvos, 2016). Systemic drug toxicities can be monitored by blood cytokine content with IL-6 and TNF-α being responsible for the majority of the observed negative effects (Tarrant, 2010). Potentially, the observed high toxicity profiles of native and designer HDP can be correlated with significantly increased pro-inflammatory cytokine production. Indeed, the oncogenic properties of the cathelin-derived peptide LL-37 are linked to its pro-inflammatory activities (Otvos and Ostorhazi, 2015). Fortunately, many other peptide families currently under preclinical studies activate the immune system by exerting anti-inflammatory responses (Otvos, 2016) culminating in potent wound-healing activities (Otvos and Ostorhazi, 2015). In our current investigation these increases do not reflect toxicity because mice treated with colistin, an antibiotic with a well-characterized toxicity profile, still produced less TNF-α and basically unchanged levels of IL-6 than their untreated counterparts. In fact, in humans, colistin significantly decreases inflammatory cytokine responses to lipopolysaccharide (LPS) exposure in blood (Matzneller et al., 2017).

It is an intriguing question why the hydrazide peptide induced a reduced level of IL-10 expression in healthy mice. In general, antibiotics, including AMP, increase anti-inflammatory cytokine production in order to control inflammation and immunopathology, but this process is antibiotic dependent. For example, while vancomycin increases IL-10 production in THP-1 monocytes, linezolid and daptomycin act in exactly the opposite way (Bode et al., 2015). When LPS are present in the medium, all 3 antibiotics induce IL-10 release, unlike cephalosporins and ciprofloxacin that reduce IL-10 expression in the presence of LPS in human blood from healthy volunteers (Ziegeler et al., 2006). Another level of complexity comes from the fact that IL-10 itself can downregulate the expression of native AMP (human β-defensin) production in certain disease conditions (Howell et al., 2005). Apparently, the connection between cytokine level changes and AMP/HDP use is highly complex and greatly depends upon the identity of the peptides, experimental conditions and other stimuli present in any given *in vitro* or *in vivo* system.

### Limitations of the cytokine profile analysis

The previous discussion topic leads us to the preliminary nature of the cytokine profile analysis upon treatment of mice with AMP/HDP. The major thrust of our paper is the comparison of the *in vivo* efficacy and toxicity parameters of *in vitro* narrow and broad spectrum peptides in order to select the optimal candidate for clinical development. With the limited analysis of cytokine expression we intended to contribute to the ever growing literature of potential alternative modes of action that ultimately make or break the ability of HDP to treat local and systemic infections. We studied only the two major anti- and two major pro-inflammatory cytokine levels; the evaluation of a comprehensive panel of cytokines is a next necessary step to fully understand the effects of HDP treatment to experimental animals and later patients. The peptides were administered im that activate muscular macrophages that are not present when the drugs are administered iv, a preferred way of therapy modality in the clinics. The study has to be expanded to a larger group size to be able to draw statistical conclusions as well as to include infected animals and bacterial breakdown products (LPS) that play roles in innate and adaptive immune reactions. Ultimately a detailed analysis of the changes and activation of immune cells upon AMP/HDP administration (Keitel et al., 2013) is a must to understand the role of immunostimulation upon peptide treatment as a therapy or prophylactic intervention.

## Concluding remarks

The current studies further support the notion that *in vitro* MIC evaluations of AMP/HDP have little prognostic value for efficacy in animal models of bacterial infections. Rather, combination of mild pro-inflammatory and strong anti-inflammatory activities suggest strong activation of the immune system of the hosts upon infection. Regarding *in vitro* toxicity evaluation, the applied peptide concentrations have to match desired TI values *in vivo*. For the specific case of amide to hydrazide conversion, from the very few published reports, one noted slightly lowered *in vivo* opioid efficacy (Wang et al., 2013) and another observed increased antinociceptive tolerance (Wang et al., 2018) suggesting less efficient turnover, two features we could corroborate in the current report. Considering that the APO-type peptides increase macrophage production in infected tissues (Ostorhazi et al., 2013) and macrophages produce high levels of IL-10 during infection (Couper et al., 2008), the combined inflammatory cytokine profile can be considered both a result and a driving force of Chex1-Arg20 amide efficacy in protecting mammals from infection and regulating host immune responses. In support of our pilot study, from anti-inflammatory cytokines macrophages do produce high IL-10 levels but not IL-4 (Cavaillon, 1994). Of particular interest, given the historic unreliability of MIC as predictor of *in vivo* activity for HDPs, there is a possibility of identifying other factors that may better correlate with this class of compounds. Cytokines, especially IL-10, may be such factors that clearly warrants further investigation.

## Author contributions

EO, RH, JW, CK, and LO: assay design, data analysis, manuscript preparation; EO: *in vivo* efficacy, toxicity, and cytokine profile studies, NH: *in vitro* MIC measurements.

### Conflict of interest statement

CK is the Chief Executive Officer of Arrevus, Inc, a biotechnology company focusing on the clinical development of the APO-type proline-arginine-rich hose defense peptides. LO is an advisor to Arrevus and is the inventor of an issued patent on the Chex1-Arg20 peptide that is licensed by Arrevus. The remaining authors declare that the research was conducted in the absence of any commercial or financial relationships that could be construed as a potential conflict of interest.

## Abbreviations

AMP: antimicrobial peptide
APO: all peptides optimized
Chex: 1-amino-cyclohexane carboxylic acid
CFU (or cfu): colony forming unit
Dab, 2: 3-diamino butyric acid
ELISA: enzyme-linked immunosorbent assay
HDP: host-defense peptide
im: intramuscularly
IL: interleukin
ip: intraperitoneally
MHB: Muller-Hinton broth
NMRI: Naval Medical Research Institute
PBS: phosphate buffered saline
RBC: red blood cells
sc: subcutaneously
TI: therapeutic index
TNF: tumor necrosis factor.
